# Recruitment strategies and reach of a digital fall-prevention intervention for community-dwelling older adults

**DOI:** 10.1177/20552076221126050

**Published:** 2022-09-15

**Authors:** Beatrice Pettersson, Saranda Bajraktari, Dawn A Skelton, Magnus Zingmark, Erik Rosendahl, Lillemor Lundin-Olsson, Marlene Sandlund

**Affiliations:** 1 Department of Community Medicine and Rehabilitation, Physiotherapy, 8075 Umeå University, Umeå, Sweden; 2 School of Health and Life Sciences, Glasgow Caledonian University, Glasgow, UK; 3 Municipality of Östersund, Health and Social Care Administration, Östersund, Sweden; 4 Department of Epidemiology and Global Health, 8075 Umeå University, Umeå, Sweden

**Keywords:** Geriatric medicine, preventive medicine, accidental falls, fall prevention, exercise, aged, eHealth, self-management, reach, recruitment

## Abstract

**Background:**

To have an impact on the population's health, preventive interventions have to reach a large proportion of the intended population. Digital solutions show potential for providing wider access to fall preventive exercise. However, there is a lack of knowledge about how to reach the target group. The aim of this study was to describe the recruitment process used in the Safe Step randomised controlled trial and the characteristics of the participants reached.

**Methods:**

Several recruitment methods, both digital and non-digital, were adopted to reach the intended sample size. Sociodemographic parameters from the baseline questionnaire were used to describe participant characteristics. The characteristics were also compared to a representative sample of older adults in the Swedish population.

**Results:**

In total, 1628 older adults were recruited. Social media proved to be the most successful recruitment strategy, through which 76% of the participants were recruited. The participants reached had a mean age of 75.9 years, lived in both urban and rural locations, were already frequent users of the Internet and applications (smartphone/tablet) (79.9%), had higher education (71.9%), and a large proportion were women (79.4%). In comparison with the general population participants in the Safe Step study were more highly educated (*p* < 0.001), women in the study more frequently lived alone (*p* < 0.001) and men more often reported poorer self-rated health (*p* = 0.04). Within the study, men reported a faster deteriorating balance (*p* = 0.003) and more prescribed medication (*p* < 0.001) than women.

**Conclusion:**

Recruitment via social media is a useful strategy for reaching older adults, especially women and frequent users of the Internet, for a fully self-managed and digital fall prevention exercise intervention. This study underlines that a range of interventions must be available to attract and suit older adults with different functional statuses and digital skills.

## Introduction

Falls are a major public health concern. Each year, about one-third of community-dwelling older adults experience at least one fall.^1^ Falls rates vary from 23% to 42% depending on the country and the age ranges considered, and about 5% of these falls results in a fracture.^2–5^ With advancing age, the risk of serious injuries or death from falls increases.^5^ Exercise as an independent intervention has proven to be particularly effective in reducing falls.^6,7^ However, more information is needed regarding how older adults can be supported to engage in and maintain an exercise behaviour.^8^ One solution is to provide interventions that can be tailored to and by the individual,^9^ and utilise older adults’ intrinsic capabilities for self-management.

In self-managed health interventions, participants have personal responsibility for their day-to-day activities and decisions,^10^ more than they might do when participating in a group or when receiving individual treatment from health care personnel. Older adults do engage in self-management of tasks to improve mobility and reduce fall accidents when sufficient information is provided at the start of the intervention, and the burden of intervention seems manageable.^11^ By supporting older adults to self-manage their own fall risk, opportunities to counteract deteriorations in balance at an early stage are created.

As a result of increased accessibility and use of digital technology among older adults,^12^ more digital solutions are being developed and tested in fall prevention trials.^13^ Several benefits are offered by implementing digital interventions, such as more options for individual tailoring, low implementation and maintenance costs, and the potential to reach and recruit a large number of participants from both rural and urban areas.^14^ In order to impact the population's health, it is important to reach out with the initiatives to a sufficient extent.^15^ Although digital solutions show potential for providing wider access to fall-preventive exercise, unsupervised exercise may not be suitable for all older adults.^16^ Some older adults may also experience difficulties when learning and managing digital technology due to impaired physical or cognitive function, lack of experience, or having limited access to the Internet.^17,18^ However, Internet access among older adults is constantly increasing. In Sweden, 9 out of 10 older adults 65 years and older report using the Internet.^18^ Daily access to the Internet via smartphone is reported by 2/3 of older adults and via tablet by 2/5.^19^

Although the possibility to reach many older adults is promising, information regarding efficient recruitment strategies for digital fall prevention interventions for older adults is lacking. Recruitment strategies for digital health interventions are often diverse and more extensive descriptions have been advocated in order to draw conclusions regarding effective strategies.^20, 21^ In addition, more knowledge regarding characteristics of older adults interested in participating in self-managed digitally delivered fall prevention exercise is of value for future implementations of fall-prevention strategies.^15^ In 2019, recruitment for the Safe Step randomised controlled trial (RCT) began on a national level in Sweden. The trial evaluates the effectiveness of a self-managed digital exercise programme, the Safe Step application, to prevent falls in older community-dwelling adults. The present study aims to describe the recruitment process and the characteristics of the participants reached.

## Methods

The Safe Step RCT study protocol, which describes the interventions in detail and the content of the Safe Step application, has been published elsewhere.^22^

### Recruitment

Participants were recruited throughout Sweden from September 2019 to April 2021. The recruitment was originally planned to last for 12 months,^22^ but was extended by 6 months to achieve enrolment of a minimum of 1400 participants. We adopted several non-digital and digital recruitment methods described below. These methods were chosen in conjunction with a spokesperson for senior citizen organisations in Sweden. The recruitment progression was continuously monitored and discussed within the research group to make adaptations, based on recruitment progression.^23^

#### Non-digital recruitment methods

Non-digital methods included advertisements in newspapers (*n* = 4), and in members’ magazines of two Swedish senior citizens organisations (*n* = 3). Two radio interviews, one national and one local were given by members of the research team. The team also introduced the study in in-person lectures given to older adults during the recruitment period (*n* = 2). To the best of our knowledge, one magazine also published an article about the study. Information about the study was sent to contact representatives (*n* = 12) of the Union of Physiotherapists, (section ‘Older adults' health’) who passed on information to other physiotherapists in their region. Information was also sent to physiotherapists working in primary health care in four regions asking them to display posters at their clinics.

#### Digital recruitment methods

Several digital recruitment methods were used. Information about the study was sent to all members in the second largest Senior citizen organisation (260,000 members) in their monthly digital newsletter. In addition, information about the study was posted on open and closed Facebook pages belonging to the Senior citizen organisations and the Union of Physiotherapist's section for older adults’ health. Paid advertisements were posted as banners on one senior organisations’ website for two separate periods of 2–3 weeks. Emails were sent to Swedish Universities for Seniors, with a request to e-mail information about the study to their participants.

Two different Facebook campaigns were used. The first ad was launched in November 2019 and was a short slideshow with text. The second ad, launched in April 2020, was a single image with text. This ad was set to be exposed only in the Facebook and Instagram news feeds. The advertisements’ performance over time was monitored, and ad spending (approximately € 20–80/day) was adjusted based on the number of recruited participants.

### Enrolment and inclusion criteria

In all our recruitment strategies, the older adults were referred to the project website (www.sakrasteg.se), where information about the study was presented in order for participants to give informed consent for participation. The information included the following: The purpose of the study, eligibility criteria, expectations of the participants in the different interventions, and guidance on how to enrol. Based on the eligibility criteria, the older adults themselves assessed whether they were suitable to participate in the study. Inclusion criteria were being 70 years or older; having fallen or experienced a decline in perceived postural balance during the last year; having access to a smartphone or tablet and using it regularly; having and using a personal email address; being able to understand verbal and written instructions in Swedish; being able to rise from a standard height chair without a person helping; and being able to walk independently indoors without a walking aid. Exclusion criteria were having a progressive disease likely to cause a decline in strength or balance over the next year; experiencing memory dysfunction that affects everyday life activities; or taking part in more than three hours per week of strenuous physical exercise causing shortness of breath.

Older adults gave informed consent for participation in the study by providing their email addresses. An email was then sent to confirm the registration and verify their email address. One reminder was sent but they were not contacted again if there was no response to this reminder. Thereafter, another email was sent with a link to the baseline questionnaire. Two automated reminders were sent, on days 7 and 14, if the questionnaire was not completed. Upon completion of the online baseline questionnaire, participants were automatically randomised by computer software in a 1:1 ratio by using permuted block randomisation.

### Measures

In the baseline questionnaire, participants reported how they first learned about the study by choosing one of six response options, or wrote their answer in an optional text field. The baseline assessment included questions about sociodemographic characteristics, postal codes, Internet use, health, and amount of physical activity and exercise. To assess the level of motivational readiness for change, the participants were asked to classify their current exercise behaviour according to the five stages of change of the Transtheoretical model.^24^

To make comparisons with a population-based cohort of adults aged 70–84 years, data was obtained from the National Public Health Survey (NPHS).^25^ The aim of the survey is to study public health in Sweden and monitor changes over time. Questions are asked regarding, for example, mental and physical health, economic conditions, and social relationships. The survey study is conducted biannually, most recently in 2018 and 2020. Each of the years, a random sample of 40,000 individuals, age 16–84, are invited to participate. In 2018–2020, 8527 older adults 70–84 years old responded to the survey, and were included as a comparison population sample in the analysis of this paper.

### Data analysis

Data analysis included a descriptive analysis of participant characteristics presented by the use of means and standard deviations for continuous data, and counts and proportions for categorical data. To compare characteristics of the participants of different recruitment paths and differences between men and women, the chi-square test of independence was performed for comparisons of nominal and ordinal variables, Fisher's Exact Test if expected count <5. For continuous variables the Mann-Whitney *U*-test was performed. For some variables, that is weight, height, and number of falls, incorrect data entries were detected and extreme outliers were removed. Cut off-values for outliers were established by expert consensus of plotted data, for age >120 (*n* = 2), weight <40 kg (*n* = 2) and >150 kg (*n* = 13), height <125 cm (*n* = 9) and >200 cm (*n* = 1), and >100 falls in 1 year (*n* = 2). Participants were excluded from specific analysis regarding falls if there were inconsistencies: multiple falls that needed medical attention (*n* = 13), or occurred indoors (*n* = 4), that exceeded their total reported number of falls. Fall rate were calculated in relation to person-years using the year participants were asked to recall as observation time.

Comparisons between the participants in the Safe Step RCT and the NPHS were analysed by Chi-square test using proportions, and unpaired *t*-test based on counts, means, and standard deviations. The statistical analyses were all performed using IBM SPSS Statistic for Windows version 26.0. *p*-values <0.05 were considered statistically significant. ArcGIS Pro software version 2.7.1 by Esri was used for analysis and visualisation as a map of the geographical distribution of the reach to participants throughout Sweden, using postal codes. Eighteen postal codes could not be verified, and are therefore not accounted for in the map.

For analysis of recruitment costs, cost-effectiveness was calculated as the cost per baseline completer, for example the total costs for advertisements in social media were divided by the number of eligible participants that completed the baseline questionnaire and stated social media as how they learned about the study. Cost were converted from the Swedish krona (SEK) to Euros (€) based on the exchange rate November 16, 2021 (10.03 SEK/€). It was not possible to distinguish costs between digital and non-digital recruitment strategies because the response categories said advertisement and did not separate banners from newspapers.

## Results

**Figure 1.**
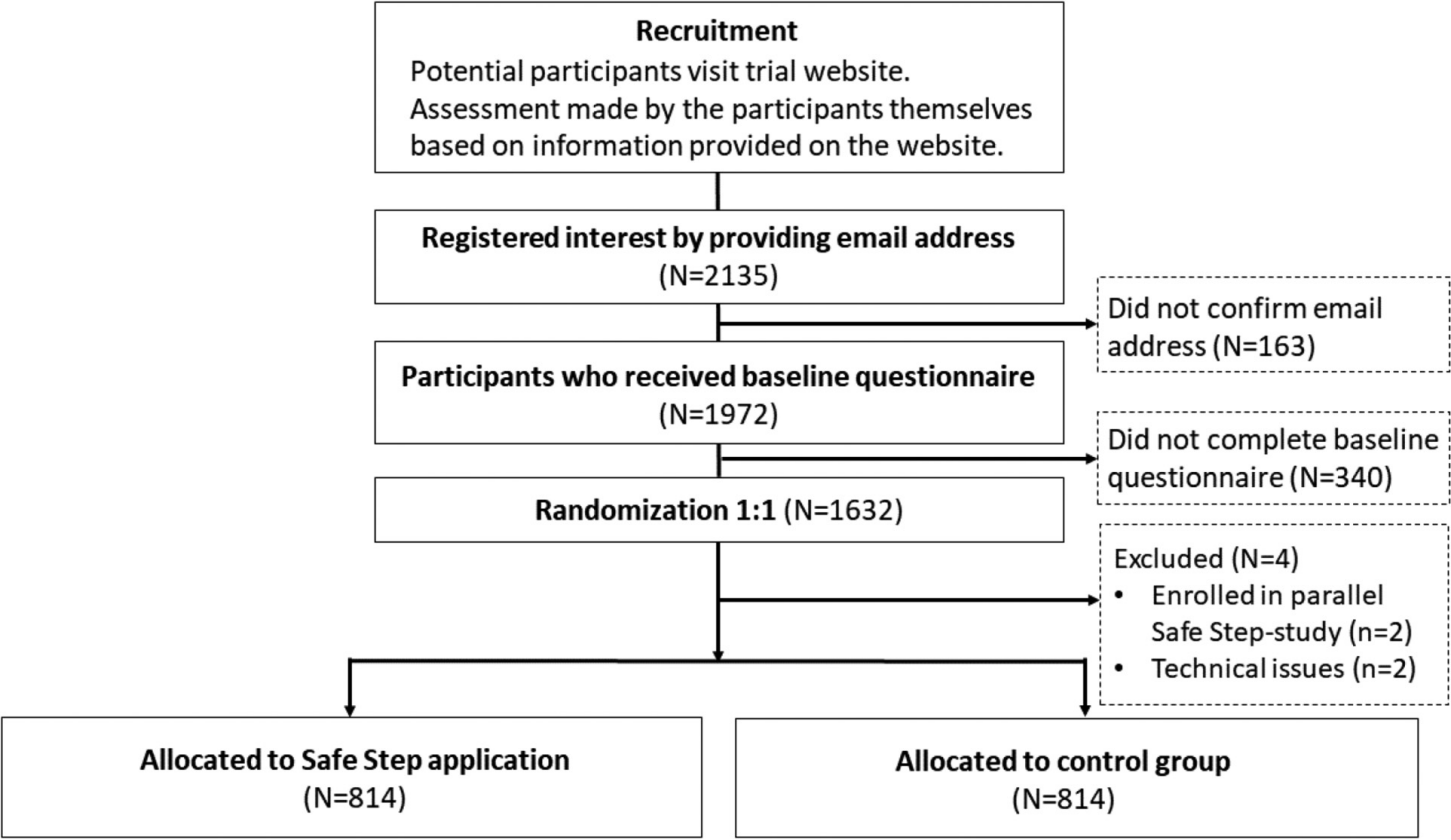
Flow chart of the recruitment process in the Safe Step randomised controlled trial (RCT).

By April 2021, 2135 individuals had registered their interest in participating in the study on the project website and 1972 had confirmed their email addresses and given informed consent to participate. In total, 1628 completed the baseline questionnaire and were subsequently randomised. A flow chart of the recruitment process is presented in Figure 1.

### Recruitment process

**Figure 2.**
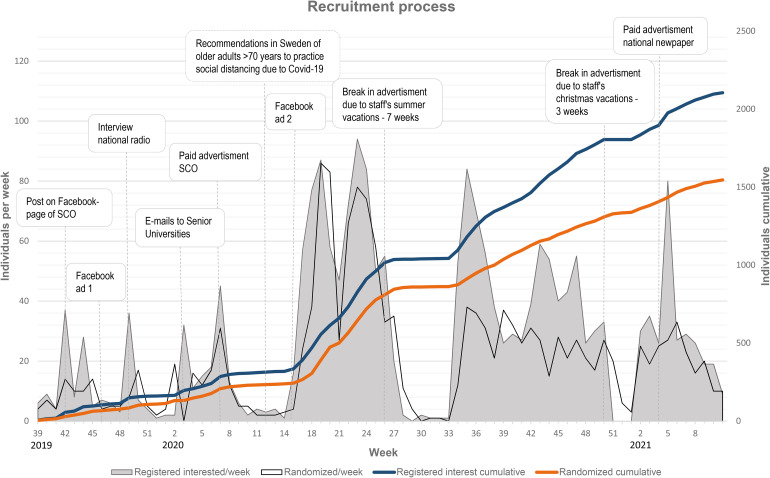
The result of the recruitment process presented by week and cumulatively over the intervention period. The weeks on the *x*-axis are presented by calender year. The text boxes represent different strategies implemented or events occurring during the intervention. SCO: senior citizen organisation.

Social media was found to be the most successful recruitment strategy, where 75.6% of the participants were recruited. The remaining participants were recruited from advertisements in newspapers (6.8%), family and friends (6.4%), senior citizen organisations (5.8%), articles in newspapers, or radio (2.3%), and other means (2.0%). Additionally, 1.0% could not remember or did not answer the question. Figure 2 illustrates the recruitment rate and implementation of different recruitment strategies. Recruitment started in September 2019, and had a mean of 8.4 recruited participants per week the first 27 weeks. During the spring of 2020, the spread of COVID-19 started to affect the general recommendations for the Swedish population. Therefore, the figure also illustrates the timepoint at which the specific recommendations for older adults over 70 years took effect. A Facebook campaign was launched that coincided with the onset of the pandemic, and thereafter the mean recruited participants per week was 27.8. The peaks observed during weeks 42–48 in 2020, can be explained by an increased amount spent on the Facebook advertisement, thus increasing the exposure. In total, the ads were exposed 2,202,854 times to 307,970 people. The ads received 51,098 clicks, of which 31,136 were unique.

### Costs

Estimates of the immediate monetary costs for advertisements was € 25,322 (€ 15,342 for ads in social media, € 9980 for ads in newspapers and senior organisation banners). In terms of cost-effectiveness, the cost per baseline completer was €15,6. Cost per baseline completer for recruitment through social media was € 12,5, and € 26,2 for participants recruited through other strategies based on newspaper and banner advertisement costs.

### Participant characteristics

The self-reported characteristics of the 1628 included participants are presented in Table 1. The mean age was 75.9 years, the majority were women who commonly used the Internet or applications on smartphones or tablets daily. The participants were prone to falls, as over half had sustained a fall in the last year, with a fall rate of 1.7 falls per person-year. The majority had more than 12 years of education, although men had a significantly lower degree of education than women. The men significantly more often reported living together with one or more persons, were more likely to have experienced a decline in balance during the last year, were more inclined to experience memory dysfunctions, and have more prescription medication per day than women (Table 1).

**Table 1.** Participant characteristics at baseline.

Variable	**Total** **(*N* = 1628)**	**Women** **(*n* = 1292)**	**Men** **(*n* = 336)**	***χ* ^2^**	***p*-value**
**Age, mean ± SD (min-max)**	75.9 ± 4.4 (70–94)	75.7 ± 4.3 (70–93)	76.9 ± 4.7 (70–94)		<0.001
**BMI, mean ± SD (*N* = 1602)**	26.7 ± 4.6	26.8 ± 4.8	26.4 ± 3.5		0.49
**Education, *n* (%)**				9.59	0.008
1–9 years	123 (7.5)	85 (6.6)	38 (11.3)		
10–12 years	335 (20.6)	262 (20.3)	73 (21.7)		
12 + years	1170 (71.9)	945 (73.1)	225 (67.0)		
**Use of Internet or applications on smart technology, *n* (%)**				3.55^a^	0.31
Multiple times per day	1154 (70.9)	921 (71.3)	233 (69.3)		
Almost every day, or at least once per week	429 (26.4)	338 (26.1)	91 (27.1)		
At least once per month but not every week, or more seldom	23 (1.4)	19 (1.5)	4 (1.2)		
Never	22 (1.4)	14 (1.1)	8 (2.4)		
** Number of fallers, *n* (%)** (*N* = 1626)	916 (56.3)	728 (56.4)	188 (56.0)	0.03	0.87
**Falls per person-year**	1.7	1.7	1.7		0.29
**Falls requiring medical attention, n (%) (*N* = 905)**	322 (12.1)	272 (12.8)	50 (9.3)	4.97	0.03
**Indoor falls, *n* (%) (*N* = 914)**	845 (31.2)	643 (30.0)	202 (36.0)	7.60	0.006
**Residency, *n* (%)**				7.26	0.03
City	1051 (64.5)	842 (65.2)	209 (62.2)		
Town	327 (20.1)	243 (18.8)	84 (25.0)		
Village or rural area	250 (15.4)	207 (16.0)	43 (12.8)		
**Household, *n* (%)**				92.89	<0.001
Live alone	728 (44.7)	656 (50.8)	72 (21.4)		
Cohabitates	900 (55.3)	636 (49.2)	264 (78.6)		
**Self-rated overall health, *n* (%)**				0.63	0.73
Very good or Good	863 (53.0)	691 (53.5)	172 (51.1)		
Fair	667 (41.0)	523 (40.5)	144 (42.9)		
Poor or Very poor	98 (6.0)	78 (6.0)	20 (6.0)		
**Prescription medications/day, *n* (%)**				32.86	<0.001
None	235 (14.4)	195 (15.1)	40 (11.9)		
1–3	779 (47.9)	655 (50.7)	124 (36.9)		
4 or more	614 (37.7)	442 (34.2)	172 (51.2)		
**Medical conditions, *n* (%)**					
Osteoarthritis	690 (42.4)	594 (46.0)	96 (28.6)	33.07	<0.001
Joints and muscle problems	624 (38.3)	523 (40.5)	101 (30.1)	12.25	<0.001
Eye disease	391 (24.0)	317 (24.5)	74 (22.0)	0.92	0.34
Dizziness	366 (22.5)	293 (22.7)	73 (21.7)	0.14	0.71
Cardiovascular disease	324 (19.9)	205 (15.9)	119 (35.4)	63.93	<0.001
Incontinence	306 (18.8)	272 (21.1)	34 (10.1)	20.89	<0.001
Osteoporosis	245 (15.0)	235 (18.2)	10 (3.0)	48.27	<0.001
Chronic lung disease	242 (14.9)	207 (16.0)	35 (10.4)	6.62	0.01
Thyroid dysfunction/metabolic disorder	216 (13.3)	204 (15.8)	12 (3.6)	34.59	<0.001
Diabetes	183 (11.2)	123 (9.5)	60 (17.9)	18.58	<0.001
Mental illness	111 (6.8)	98 (7.6)	13 (3.9)	5.80	0.02
**Experiences of memory dysfunction, *n* (%)**				23.40	<0.001
Yes, affects everyday life	67 (4.1)	43 (3.3)	24 (7.1)		
Yes, does not affect everyday life	699 (43.0)	530 (41.0)	169 (50.3)		
No	862 (52.9)	719 (55.7)	143 (42.6)		
**Gait speed compared to peers, *n* (%)**				1.31	0.52
Faster	397 (24.4)	322 (25.0)	75 (22.4)		
As fast	557 (34.2)	443 (34.3)	114 (33.9)		
Slower	674 (41.4)	527 (40.7)	147 (43.7)		
**Perceived balance, *n* (%)**				0.01	1.00
Very good or good	268 (16.5)	213 (16.5)	55 (16.4)		
Fair	821 (50.4)	652 (50.5)	169 (50.3)		
Poor or very poor	539 (33.1)	427 (33.0)	112 (33.3)		
**Perceived change in balance, last year, *n* (%)**				11.65	0.003
Better	62 (3.8)	49 (3.8)	13 (3.9)		
The same	787 (48.3)	652 (50.5)	135 (40.2)		
Worse	779 (47.9)	591 (45.7)	188 (55.9)		
**Perceived leg strength, *n* (%)**				0.80	0.67
Very good or good	584 (35.9)	460 (35.6)	124 (36.9)		
Fair	703 (43.2)	565 (43.7)	138 (41.1)		
Poor or very poor	341 (20.9)	267 (20.7)	74 (22.0)		
**Walking aid, *n* (%)**	297 (18.2)	229 (17.7)	68 (20.2)	1.13	0.29
**Physical activity, *n* (%)**					
>2 h/week physical daily activities	523 (32.1)	413 (32.0)	110 (32.7)	0.11	0.79
>2 h/week strenuous physical activities	115 (7.1)	80 (6.2)	35 (10.4)	8.82	0.007
**TTM, *n* (%)**				1.31	0.86
Maintenance	832 (51.1)	658 (50.9)	174 (51.8)		
Action	132 (8.1)	102 (7.9)	30 (8.9)		
Preparation	175 (10.8)	144 (11.1)	31 (9.2)		
Contemplation	297 (18.2)	236 (18.3)	61 (18.2)		
Precontemplation	192 (11.8)	152 (11.8)	40 (11.9)		

χ^2^: Chi-square; BMI: body mass index; TTM: transtheoretical model; Precontemplation: not engaging in regular exercise and no intention to start in the future; Preparation: seriously considering to start exercising – has taken some steps toward the objective; Maintenance: exercising consistently for 6 months or more, ^a^Fischer's exact test.

Participants recruited through social media were more likely to be women χ^2^ (38.63, *df* *=* *1*, *p* *<* 0*.*001), more highly educated χ^2^ (10.57, *df* *=* *2 p* *=* 0.005) and more likely to use the Internet or apps on smart technology more frequently χ^2^ (22.74, *df* *=* *3, p* *<* 0.001) compared to those recruited by other strategies. Participants recruited through social media also more often reported living in less populated communities χ^2^ (6.28, *df* *=* *2, p* *=* 0.04) (Table 1).

**Figure 3.**
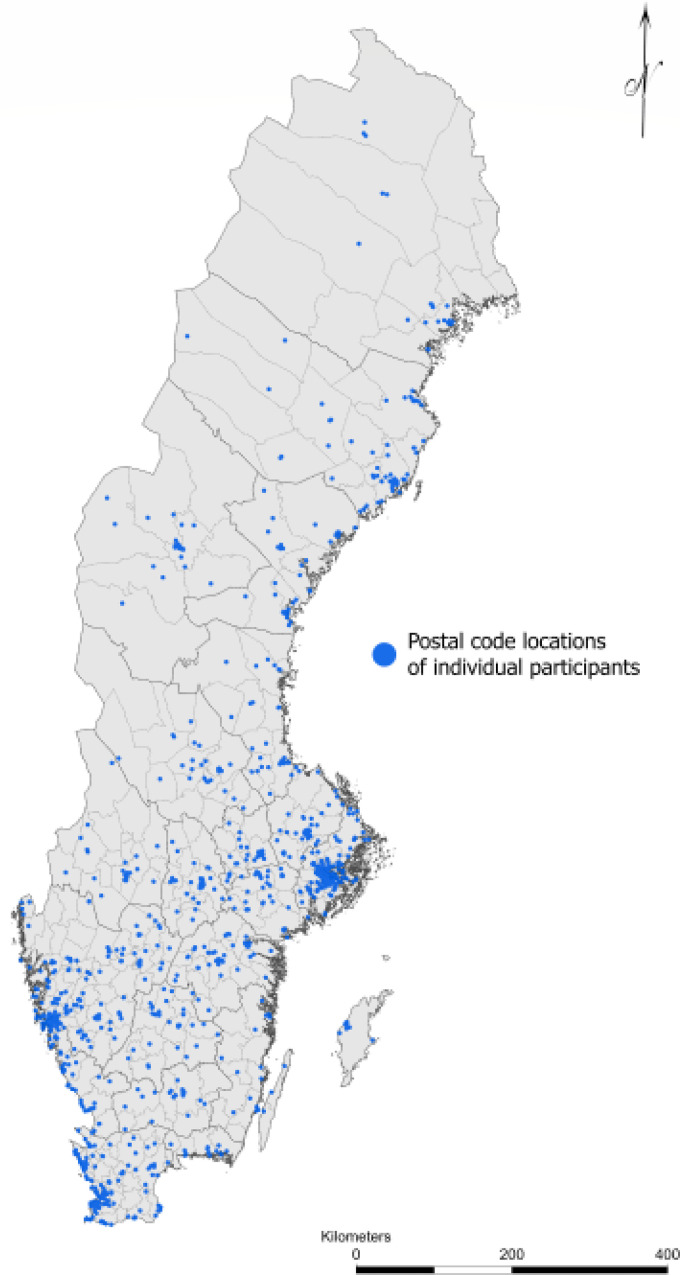
Illustration of the participant recruitment throughout Sweden.

Participants were recruited throughout Sweden and the distribution shown in Figure 3 is reflective of the national demographic distribution, with a greater proportion living in southern and coastal regions. Approximately two-thirds of study participants reported living in cities.

### Representativeness

Participants recruited for this study had a significantly higher degree of education and more frequently lived alone than the older adults who responded to the NPHS in 2018 and 2020 (Table 2). Women in the Safe Step study more frequently lived alone (*n* = 656, 50.8%), than in the NPHS cohort (*n* = 1858, 42.9%), χ^2^ (25.09, *df* *=* *1, p* < 0.001) while the men were representative of the male population (*n* = 72, 21.4%/*n* = 940, 22.4%, χ^2^ (0.17, *df* *=* *1*, *p* *=* *0*.*68*)). No difference in self-rated health was found between the two cohorts, however in a stratified analysis men reported better self-rated health in the NPHS-cohort (*n* = 2350, 57.0%) than participants in the Safe Step project (*n* = 172, 51.1%) χ^2^ (4.33, *df* *=* *1*, *p* *=* 0.04), but not women (*n* = 2270, 54.0%/*n* = 691, 53.0%), χ^2^ (0.14, *df* *=* *1*, *p* *=* 0.70).

**Table 2.** Comparison between participant in the Safe Step RCT and the general Swedish population (NPHS).

Variable	Safe step	**NPHS**	**χ^2^**	***p*-value**
	(*N* = 1628)	(*N* = 8527)		
**Age, mean ± SD (min-max)**	75.9 ± 4.4 (70-94)	76.0 ± 3.5 (70-84)		0.31
**Women, *n* (%)**	1292 (79.4)	4333 (50.8)	501.17	<0.001
**Living alone, *n* (%)**	728 (44.7)	2798 (32.8)	85.46	<0.001
				
**Education, *n* (%)**		(*N* = 8476)		
1–9 years	123 (7.5)	4765 (56.2)	2101.72	<0.001
10–12 years	335(20.6)	2131 (25.1)		
12 + years	1170 (71.9)	1580 (18.6)		
		(*N* = 8362)		
**Very good or** g**ood self-rated overall health, *n* (%)**	863 (53.0)	4642 (55.5)	3.45	0.06
	(*N* = 1602)	(*N* = 8181)		
**BMI ≥30, *n* (%)**	318 (19.5)	1476 (18.0)	2.93	0.09

The selection of participants from the NPHS was adults aged 70–86 years. NPHS, National Public Health Survey, χ^2^, Chi-square, BMI, body mass index.

## Discussion

We found that using social media was an efficient approach for the recruitment of older adults for a digital intervention. It attracted mostly highly educated women but also attracted men with lower self-rated health, and more health issues and medications. In addition, the costs for recruitment through social media were markedly lower than the cost for advertisements in newspapers and banners. Older adults were recruited and randomised across Sweden, with a good spread of socio-demographic factors and geographical representation.

The success of using social media for participant recruitment is in accordance with a systematic review that concluded that Facebook advertisement was more efficient and less expensive, in comparison with traditional recruitment methods, when recruiting younger participants in health research.^14^ However, studies on the recruitment of older adults through social media are scarce. We used social media for advertisement over two separate periods. The first advertisement was launched in November 2019, but we did not see a substantial increase in recruited participants. In April 2020, we launched a second advertisement, which led to a large increase in the number of recruited participants. During that time, the global pandemic of COVID-19 had started to lead to social restrictions to avoid the spread of the disease. Therefore, we can assume that the increased number of interested participants during the second campaign was facilitated by the COVID-19 pandemic. Supporting this assumption is the increase in the use of Facebook and Instagram among Swedish older adults during the pandemic, where over two-thirds of adults over 76 years of age used Facebook and one-third used Instagram in late 2020.^18^ However, we also made an alteration to the Facebook campaign, from a series of pictures to one picture, and alterations were also made to the text and placement of the ads. Therefore, no distinct conclusion can be drawn regarding what was the greatest contributing factor to the large increase of recruited participants experienced during spring 2020.

We reached older adults living both in cities and in rural areas. The majority of our recruited participants were women, which may be due to the high success rate of recruitment via social media, where women are more represented.^18^ Sampling bias may also have occurred due to Facebook's targeting algorithm. The algorithm learns from interactions with the ad and shows the ad to people with similar profiles. However, the disposition of more women in the trial shows similarity with traditional fall prevention trials.^6^ Indeed, in a qualitative study, women expressed themselves as more likely to be receptive to fall-prevention information and were seen by the men as in more need of fall-prevention measures. The men also expressed a need to be motivated from women/their spouses to sign up for health promotion programmes.^26^ By way of confirmation, in our study, men were more often cohabitating than women. Interestingly, men also significantly less often rated their health as good or very good compared to the men in the NPHS and had more health issues and medications compared to the women in the RCT. Gender differences in fall prevention research require further investigation as few studies address these issues.^27^

We found that the participants enrolled in the Safe Step RCT were more highly educated in comparison with their peers in the general population in Sweden. Not surprisingly, the recruitment strategies and the fully self-managed design of the Safe Step RCT also seem to attract older adults accustomed to using the Internet and applications on smartphones and tablets. It, therefore, seems likely that the study attracted older adults with high self-efficacy for seeking information and managing their health through online resources, thus having high eHealth literacy.^28,29^ Therefore, the nature of the study might present issues of digital exclusion, as many older adults might not have access to the technology, have cognitive impairments, lower educational level, or not have the confidence or skill to use it.^30^ In a municipality-based observational study, the Safe Step application was complemented with optional supportive strategies that had been co-created with older adults. These strategies included introductory drop-in meetings, group exercise sessions, and in-person technical support. Nevertheless, the interest in the supportive strategies were low.^31^ Additional strategies are therefore needed to attract older adults who are less prone to using digital technology for health purposes. In a previous study, we found that a proportion of people did not wish to use digital technology and instead used paper-based resources,^32^ but it is likely that more regular prompting to support behaviour change and regularity of use may be necessary as compliance to the exercise prescription was better when prompted by the digital technology.^32^ Although eHealth literacy is negatively associated with age,^28^ and older adults are more often represented among non-users of the Internet,^18^ this group is rapidly decreasing, and older adults are becoming more accustomed to using smartphones and tablets.^18^ Therefore, the potential to reach older adults with digitally administered fall prevention strategies seems promising. However, as also indicated by the results of the present study, this mode of delivery of fall prevention exercise will not suit all older adults and should be seen as one solution among many.

Health care across the globe will have to undergo structural changes to meet the demands of the growing and ageing population. We have to provide a variety of solutions adaptable to older adults’ different preferences regarding engagement with fall prevention interventions.^9,27^ The results of the present study suggest that self-managed digital fall prevention without interaction with a health care provider could be one solution to reach older adults with better functional status, who might not visit health care on a regular basis. As can be seen in this study, this group of older adults is still prone to falls.

### Methodological discussion

A strength of this study includes the significant number of participants recruited, the randomised design, and the limited interaction with the participants. All of which contribute to more generalisable results.

We saw some discrepancies between the self-assessed inclusion criteria and the responses in the baseline questionnaire; Although participants were informed that memory impairment affecting their daily lives constituted grounds for exclusion, 4.1% stated that this was the case for them. The inclusion criteria also specified experiences of a fall event or perceived balance deficiency in the last year, of which 21.4% of the participants reported neither. Nevertheless, participants may still have experienced a reduced balance developed over recent years.

During the recruitment process, we encountered some difficulties sending automated emails to the participants, as we discovered that emails sent to certain domains were marked as spam and never received by participants. Therefore, a personalised email from study staff was sent to participants who had not confirmed their email addresses. For the same reason, a personalised email was also sent to those who had not answered the baseline questionnaire during the last five months of the study.

Another strength of the study is the comparison of the participants in the Safe step RCT with the respondents to the NPHS. However, the comparison shows some limitations as their selection from the general population excludes adults above age 84 and the distribution of men and women does not reflect the population in general among older adults.^25^

A limitation of the study is that we attracted technology-literate, well-educated white women. Technology use often differs across ethnic groups and language barriers can also affect inclusion and participation.

## Conclusion

Social media was a more efficient and cost-effective approach than non-digital methods for recruiting older adults for a digital fall prevention exercise intervention. We reached participants all over Sweden, both in rural and urban locations. A fully self-managed digital fall prevention intervention seems to attract more women, those with higher education, and who are frequent users of Internet and applications on their smartphone or tablet, but also men who had more health issues, medications, and were more likely to consider their balance as deteriorating more quickly. The results of this study further emphasise that a range of interventions need to be available to attract and suit as many older adults as possible to engage in fall prevention exercise.
